# Macroprolactinemia in patients with hyperprolactinemia: an experience from a single tertiary center

**DOI:** 10.11604/pamj.2020.36.8.22923

**Published:** 2020-05-06

**Authors:** Phronpan Chutpiboonwat, Kanchana Yenpinyosuk, Vitaya Sridama, Supaksorn Kunjan, Karuna Klaimukh, Thiti Snabboon

**Affiliations:** 1 Department of Medicine, Bangkok Metropolitan Administration Lat Krabang Hospital, Bangkok, Thailand; 2 Department of Medicine, Faculty of Medicine, Chulalongkorn University, Bangkok, Thailand; 3 Saint Louis Hospital, Bangkok, Thailand; 4 Center for Medical Diagnostic Laboratories, Faculty of Medicine, Chulalongkorn University, King Chulalongkorn Memorial Hospital, Bangkok, Thailand; 5 Excellent Center in Diabetes, Hormone and Metabolism, King Chulalongkorn Memorial Hospital, Thai Red Cross Society, Bangkok, Thailand

**Keywords:** Macroprolactinemia, hyperprolactinemia, polyethylene glycol precipitation

## Abstract

Macroprolactinemia frequently causes misdiagnosis, unnecessary investigation and inappropriate treatment in hyperprolactinemic patients. Aim of this study is to investigate the prevalence and clinical characteristics of Thai patients with macroprolactinemia. We performed a cross-sectional study in 56 hyperprolactinemic patients (51 women and 5 men) whose sera were subsequently tested for the presence of macroprolactin. Recovery of less than 40% of serum prolactin after polyethylene glycol (PEG) precipitation was indicative of macroprolactinemia. Our study revealed 19.64% (11/56) of patients with hyperprolactinemia were found to have a preponderance of macroprolactin. All patients with macroprolactinemia were women, of which eight of them were initially diagnosed as idiopathic hyperprolactinemia and mistreated with dopamine agonist medications. Interestingly, neuroradiological abnormalities were reported in three patients with macroprolactinemia, 2 cases with prolactinoma and one case with stalk effect hyperprolactinemia. In conclusion, nearly one-fifth of our patients with hyperprolactinemia have macroprolactinemia. This finding suggests that the diagnostic algorithm of all patients with hyperprolactinemia should include the PEG precipitation test as the initial step.

Domain: Endocrinology

## Introduction

Hyperprolactinemia is a common endocrine problem in general practice. Clinically, it manifests mainly as reproductive and sexual dysfunction including amenorrhea or oligomenorrhea, galactorrhea and infertility in women or loss of libido in men [1, 2]. Etiologies of hyperprolactinemia can be classified into three categories: physiologic, pharmacologic, and pathologic. Prolactin (PRL) circulates in human sera in three major forms according to their molecular size; monomeric (23 kDa), big or dimeric (45-60 kDa) and big-big (150-170 kDa) or macroprolactin. Monomeric form is the most prominent form (85-95%) of the circulating PRL and is known to be biologically and immunologically active. Macroprolactin accounts for less than 5% of circulating PRL and can interfere with all currently available commercial PRL immunoassays leading to falsely elevated PRL levels in terms of macroprolactinemia1. Several recommendations include the determination of macroprolactin as the first in the differential diagnosis of hyperprolactinemia [2-5]. A simple and inexpensive method using polyethylene glycol (PEG), precipitation has been accepted as a screening test to identify the presence of macroprolactin in serum instead of gel filtration chromatography which is too time-consuming and expensive [6, 7]. The aim of this study is to investigate the prevalence of macroprolactinemia in hyperprolactinemic Thai patients by using PEG precipitation, and to determine the clinical and neuroradiological features of affected individuals.

## Methods

Patients with hyperprolactinemia or PRL levels of more than 25 ng/mL were followed by the Endocrine Clinic, King Chulalongkorn Memorial Hospital, Chulalongkorn University, Bangkok, Thailand were retrospectively evaluated. Patients with lactation or pregnancy were excluded. Their sera were centrifuged and stored at -80°C for subsequent determination of macroprolactin. Complete medical data of the patients were reviewed including presenting signs and symptoms related to hyperprolactinemia such as menstrual irregularities, infertility or galactorrhea in women or impotence in men, the presence of headache and visual loss, physical examination, hormonal and imaging study and their treatment. Imaging data were studied by maximum diameter and invasion of tumor from magnetic resonance imaging (MRI) study. Tumor size was classified as microadenoma (< 1 cm) and macroadenoma. The study was approved by the Ethical Committee of Chulalongkorn University and written informed consent was obtained from each subject. Serum PRL was measured by the Abbott Architect LN 7K76 prolactin chemiluminescent microparticle immunoassay (Abbott Laboratories, USA) on an Abbott Architect i2000 SR platform. PEG precipitation tests were used as method for detection of macroprolactin [8]. In brief, to perform PEG precipitation, equal volumes (200 μL) of a 25% solution of PEG (molecular weight 6,000 kDa) and patient´s serum were mixed and centrifuged at 1,500g for 30 min. Immunoreactive PRL was measured in the supernatant, and the results after correction for dilution were compared with those obtained from unprecipitated serum. The results were expressed as the percent of PRL recovered. Recovery less than or equal to 40% of initial PRL value was taken as evidence that a significant level of macroprolactin was present in the serum [7, 8]. The within-assay and inter-assay CV for PRL recoveries were 2.7% and 3.7%, respectively.

**Statistical analyses:** the data was analyzed on SPSS (version 17.0, Chicago, IL, USA). Patients were stratified under macroprolactinemia group and true or monomeric hyperprolactinemia group, according to monomeric reference range after PEG treatment. Frequencies and percentages for categorical variable, mean and standard deviation (SD) for discrete or continuous variables and for non-normal distribution data with median values were calculated.

## Results

Fifty-six consecutively hyperprolactinemic patients (5 men and 51 women, aged 20-69 years; mean age 38.64 years) were recruited. Their serum PRL levels in untreated sera ranged from 35.5 to 7.350 ng/mL. Clinical characteristics of the patients are shown in Table 1. Menstrual abnormalities and galactorrhea were common presentations in women while mass effect and loss of libido were most common in male patients. Prolactinoma (37 cases) and idiopathic hyperprolactinemia - hyperprolactinemia with normal imaging study (10 cases) were common etiologies among our patients with hyperprolactinemia. In addition, all male patients with prolactinoma had macroadenoma. Macroprolactinemia was found in 11 out of 56 (19.64%) hyperprolactinemic patients and all were female. Isolated macroprolactinemia was commonly found in idiopathic hyperprolactinemia who presented only menstrual abnormalities (Table 2). Initial serum PRL in macroprolactinemia group ranged between 37.4 and 7.350 ng/mL and nine of them returned to normal levels after PEG precipitation. Magnetic resonance imaging (MRI) of the pituitary gland was abnormal in 3 of 11patients. Two patients were found to have prolactinoma, while the other had a meningioma (Table 3). Interestingly, all patients with macroprolactinemia which were initially diagnosed as idiopathic hyperprolactinemia and treated with dopamine agonist medications.

**Table 1:** Clinical and laboratory characteristics of patients with monomeric hyperprolactinemia and macroprolactinemia

Characteristics	Monomeric Hyperprolactinemia (n = 47)*	Macroprolactinemia (n = 11)*
Gender (M : F)	5 : 42	0 : 11
Median age (range, years)	39 (20-69)	35 (22-67)
Total PRL (range, ng/mL)	35.5-7,310	37.4-7,350
PRL after PEG precipitation (range, ng/mL)	28.7-6,105	2.6-821
Clinical features (case/total)		
Menstrual abnormalities (F)	38/42	11/11
Galactorrhea (F)	26/42	3/11
Loss of libido (M)	5/5	0/0
Headache	10/47	0/11
Visual field defects**	12/47	0/11
Imaging abnormality (case/total)	40/47	3/11

* Two patients had both elevated monomeric PRL and macroprolactin levels

** Nine from ten patients with macroprolactinoma and all patients with hyperprolactinemia from stalk effect

**Table 2:** Patient characteristics of the different diagnostic groups with macroprolactinemia

Etiologies of Hyperprolactinemia	Total number	Median age (years)	Median serum PRL (ng/mL)	Number of Macroprolactinemia
Idiopathic	10	35	56.3	8
Microprolactinoma	27	39	81.8	1
Macroprolactinoma	10	45	251.4	1
Stalk compression	3	37	57.7	1
Drug-induced	6	29	45.3	0

**Table 3:** Clinical characteristics of the patients with macroprolactinemia

Case	Sex	Age (y)	Presenting symptoms	MRI of the pituitary	PRL levels (PEG precipitation) (ng/mL)		
					before	after	% recovery
I	F	47	headache, visual filed defect	sphenoid meningioma	82.9	21.8	26.29
II	F	30	infertility	normal	42.4	6.4	15.09
III	F	40	amenorrhea	normal	56.3	7.2	12.79
IV	F	34	infertility	normal	64.05	2.6	4.06
V	F	23	amenorrhea	normal	37.4	7.7	20.59
VI	F	22	amenorrhea, galactorrhea	microprolactinoma	253.2	36.2	14.29
VII	F	22	infertility	normal	85	11	12.94
VIII	F	35	amenorrhea	normal	74.5	4.6	6.17
IX	F	36	amenorrhea	normal	56	5.4	9.64
X	F	67	headache, visual filed defect	macroprolactinoma	7,350	821	11.17
XI	F	37	amenorrhea	normal	66.7	6.2	9.29

## Discussion

PRL measurement is one common laboratory test used in clinical practice, particularly in the reproductive field [5]. Errors in PRL measurement from falsely lowered levels or hook effect and falsely elevated levels or macroprolactinemia result in unnecessary investigation and inappropriate treatment in hyperprolactinemic patients [1,9]. Our study demonstrates about at least one-fifth of hyperprolactinemic patients have misdiagnosis from the presence of macroprolactinemia. Macroprolactin is a heterogeneous complex form of monomeric PRL binding to IgG and other non-IgG substances, when it increases more than 40 or 60% total circulating PRL is called macroprolactinemia. Its clinical significance is believed to biological inactivity in vivo because it cannot cross the endothelial lining and reach target organs [4]. Therefore, patients with macroprolactinemia should not manifest signs and symptoms of hyperprolactinemia. However, with non-specific symptoms such as menstrual irregularity coupled with easy access to perform PRL measurement, there will be a coincidence of such symptoms and the presence of macroprolactinemia as seen in patients with idiopathic hyperprolactinemia. Concordantly, data of prevalence in Caucasian and Asian subjects ranged from 10% to 62% [8-11]. The differences in the prevalence of macroprolactinemia in other studies can be explained by the variable cutoff levels of recovery indicative of macroprolactinemia and the assay used to measure PRL. Even though our study had limit in number, it confirms that clinical manifestations of patients with macroprolactinemia cannot be differentiated from those with true or monomeric hyperprolactinemia, except through the macroprolactin measurement. In addition, the co-incidence between both macrorprolactinemia and monomeric hyperprolactinemia were noted and similar to previous reports as 7-20% of macroprolactinemic patients [10]. Therefore, the presence of macroprolactinemia does not exclude the possibility of pituitary pathology particularly in case of post PEG PRL level above the normal reference range. Currently, The Endocrine Society guidelines recommend screening for macroprolactinemia in all asymptomatic patients with elevated PRL levels [5]. There are two ways to report the presence of macroprolactinemia including PRL recovery (% recovery, %) and PRL concentration after PEG treatment (post PEG PRL, ng/mL). Nowadays, it is recommended to report results as post PEG PRL using post-PEG reference ranges; however, normal reference ranges of post PEG PRL are still required to be established.

## Conclusion

Our study reveals that nearly 20% of our patients with hyperprolactinemia have macroprolactinemia. This finding supports the inclusion of PEG precipitation in the diagnostic algorithm of patients with hyperprolactinemia to preclude unnecessary further investigations and treatment.

### What is known about this topic

- Clinically, hyperprolactinemia manifests mainly as reproductive and sexual dysfunction including amenorrhea or oligomenorrhea, galactorrhea and infertility in women or loss of libido in men;
- Errors in PRL measurement from falsely lowered levels or hook effect and falsely elevated levels or macroprolactinemia result in unnecessary investigation and inappropriate treatment in hyperprolactinemic patients;
- Macroprolactin can interfere with all currently available commercial PRL immunoassays leading to falsely elevated PRL levels in terms of macroprolactinemia.

### What this study adds

- Our study demonstrates about at least one-fifth of hyperprolactinemic patients have misdiagnosis from the presence of macroprolactinemia;
- Co-incidence between both macrorprolactinemia and monomeric hyperprolactinemia were noted; therefore, the presence of macroprolactinemia does not exclude the possibility of pituitary pathology particularly in case of post PEG PRL level above the normal reference range.

## Competing interests

The authors declare no competing interests.
